# Association of red blood cells and plasma transfusion versus red blood cell transfusion only with survival for treatment of major traumatic hemorrhage in prehospital setting in England: a multicenter study

**DOI:** 10.1186/s13054-022-04279-4

**Published:** 2023-01-17

**Authors:** Harriet Tucker, Karim Brohi, Joachim Tan, Christopher Aylwin, Roger Bloomer, Rebecca Cardigan, Ross Davenport, Edward D. Davies, Phillip Godfrey, Rachel Hawes, Richard Lyon, Josephine McCullagh, Simon Stanworth, Julian Thompson, James Uprichard, Simon Walsh, Anne Weaver, Laura Green

**Affiliations:** 1 Centre for Trauma Sciences, Blizard Institute, Queen Mary University of London, 4 Newark Street, London, E1 2AT UK; 2 Barts Health NHS Trust, London, UK; 3 St George’s University of London, London, UK; 4 St Mary’s Hospital, Imperial College NHS Foundation Trust, London, UK; 5 Kings College Hospital NHS Foundation Trust, London, UK; 6 NHS Blood and Transplant, Cambridge, UK; 7 Royal Preston Hospital, Preston, UK; 8 James Cook University Hospital, Middlesbrough, UK; 9 Newcastle Upon Tyne NHS Foundation Trust, Newcastle, UK; 10 Great North Air Ambulance, Stockton-on-Tees, UK; 11 Air Ambulance Kent Surrey Sussex, Kent, UK; 12 Oxford University Hospital NHS Foundation Trust, Oxford, UK; 13 Southmead Hospital, Bristol, UK; 14 Great West Air Ambulance, Bristol, UK; 15 St George’s University Hospital NHS Foundation Trust, London, UK; 16 Essex and Hertfordshire Air Ambulance Trust, Essex, UK

**Keywords:** Trauma, Prehospital transfusion, Combined red cell and plasma, Mortality

## Abstract

**Background:**

In-hospital acute resuscitation in trauma has evolved toward early and balanced transfusion resuscitation with red blood cells (RBC) and plasma being transfused in equal ratios. Being able to deliver this ratio in prehospital environments is a challenge. A combined component, like leukocyte-depleted red cell and plasma (RCP), could facilitate early prehospital resuscitation with RBC and plasma, while at the same time improving logistics for the team. However, there is limited evidence on the clinical benefits of RCP.

**Objective:**

To compare prehospital transfusion of combined RCP versus RBC alone or RBC and plasma separately (RBC + P) on mortality in trauma bleeding patients.

**Methods:**

Data were collected prospectively on patients who received prehospital transfusion (RBC + thawed plasma/Lyoplas or RCP) for traumatic hemorrhage from six prehospital services in England (2018–2020). Retrospective data on patients who transfused RBC from 2015 to 2018 were included for comparison. The association between transfusion arms and 24-h and 30-day mortality, adjusting for age, injury mechanism, age, prehospital heart rate and blood pressure, was evaluated using generalized estimating equations.

**Results:**

Out of 970 recruited patients, 909 fulfilled the study criteria (RBC + P = 391, RCP = 295, RBC = 223). RBC + P patients were older (mean age 42 vs 35 years for RCP and RBC), and 80% had a blunt injury (RCP = 52%, RBC = 56%). RCP and RBC + P were associated with lower odds of death at 24-h, compared to RBC alone (adjusted odds ratio [aOR] 0.69 [95%CI: 0.52; 0.92] and 0.60 [95%CI: 0.32; 1.13], respectively). The lower odds of death for RBC + P and RCP vs RBC were driven by penetrating injury (aOR 0.22 [95%CI: 0.10; 0.53] and 0.39 [95%CI: 0.20; 0.76], respectively). There was no association between RCP or RBC + P with 30-day survival vs RBC.

**Conclusion:**

Prehospital plasma transfusion for penetrating injury was associated with lower odds of death at 24-h compared to RBC alone. Large trials are needed to confirm these findings.

**Supplementary Information:**

The online version contains supplementary material available at 10.1186/s13054-022-04279-4.

## Background

The majority of deaths from traumatic hemorrhage occur within the first three hours of injury [1]. This is often prehospital, especially in the more rural, remote or austere settings. While in-hospital acute resuscitation in trauma has evolved toward early and balanced resuscitation with red blood cells (RBC), plasma, and platelets being transfused in equal ratios [2, 3, 4], this is challenging to deliver prehospital. In prehospital environments, the logistics of delivering several blood components could be challenging as this would necessitate carrying additional blood storage boxes for the prehospital personnel and increase the complexity of resuscitating patients due to several bags needing to be administered to patients (who may not have enough intravenous access) and delay their transfer to hospital.

Several military and civilian observational studies have reported survival benefits with prehospital RBC transfusion, with the effect being greater if transfusion was started within 15 min [1, 5, 6]. However, we need to acknowledge the risk of selection bias from these study designs. In contrast, a recent randomized controlled trial (RCT) showed that RBC transfusion plus lyophilized plasma in the prehospital setting was not superior to saline resuscitation for improving tissue perfusion or reducing episode mortality; however, at 3-h, the adjusted average difference in mortality was 7% lower (95% CI: 15% lower to 1% higher, *p* = 0.08) in the blood components arm [7]. Two RCTs (PAMPer and COMBAT) evaluated the role of plasma transfusion in prehospital resuscitation and showed conflicting results [8, 9], although their combined analysis indicated that the survival benefit is greatest in patients who received both RBC and plasma, had blunt injuries [10], and longer prehospital transport times (> 20 min) [10, 11, 12, 13]. The evidence on the effect of prehospital plasma transfusion in addition to RBC transfusion versus RBC transfusion alone is limited.

The drive to deliver an early 1:1 ratio of RBC and plasma transfusion early has led to an increasing interest in the use of a whole blood (WB) component based on military experience [14], even though evidence on its benefits and risks is limited [15, 16, 17]. In the United Kingdom (UK), most prehospital services carry RBC and thawed plasma. Due to storage requirements, it is not possible to carry platelets in the prehospital setting. Currently, in the UK, the leukocyte depletion process introduced in the 1990s to reduce the risk of variant CJD transmission via blood transfusion removes 80% of platelets in whole blood donations, and therefore, the remaining component contains red cells and plasma in one bag (herein referred to as RCP) [18]. The prehospital use of the RCP component in traumatic hemorrhage may offer logistical and clinical benefits compared to the use of separate blood components; however, this has not been evaluated before.

The overall objective of this multicenter observational cohort study was to evaluate the effect of prehospital RCP transfusion (RCP arm) on 24-h mortality and 30-day mortality in traumatically injured bleeding patients, when compared with prehospital RBC transfusion only (RBC arm) and prehospital RBC plus plasma transfusion (RBC + P arm).

## Methods

The study received ethical approval from the UK Health Research Authority (IRAS reference: 236783). Data were collected from six Helicopter Emergency Medical Services (HEMS) in England. All patients underwent their normal course of treatment as directed by their HEMS and hospital protocols and at no point was their care altered for the purpose of this study. Data for the RCP and RBC + P arms were collected prospectively between October 2018 and October 2020, while data for the RBC arm were collected retrospectively between October 2015 and October 2018.

### Study setting

The participating HEMS in this study all operate within their regional major trauma systems (MTC). The HEMS teams can transfer traumatically injured bleeding patients directly from scene to the regional MTC. A total of nine MTCs participated in this study, four within the London region and five outside. HEMS that work across MTCs may transfer patients to more than one MTC; conversely, each MTC may receive trauma patients from more than one HEMS. The HEMS operate across rural and urban settings with access to both air and road transport modalities dependent on time of day, weather and operational constraints. Operational models were doctor-paramedic, or doctor-doctor-paramedic in 5 services, with one service running a two critical care paramedic model for 20% of operational hours, during which blood transfusion was authorized by an on-call consultant.

### Study population

Patients were included if they had suffered traumatic injury, were attended by a HEMS team and had started receiving or received at least one type of blood component or blood products (i.e., Lyophilized plasma or Lyoplas) prehospital, according to local protocols. All age groups were included. Patients were excluded from the main analysis if they were transfused for non-traumatic indications and/or if they were admitted to hospital from another hospital rather than HEMS (or secondary transfer). All patients were followed up for 30 days or until death, whichever occurred first.

### Interventions

The RBC arm carried four units of RBC (each ~ 250 mL) and data for this arm were from one service (London Air Ambulance) only. The RCP arm carried two units of RCP (each 470 mL). A maximum of up to four RCP units could be given to any patient. The RCP units were derived from whole blood donations that were leucocyte-depleted and collected into 66.5 mL CPD following standard operating procedures. RCP units are stored in the same way as the red blood cells [18]. The RBC + P arm transfused two units of RBC and two units of thawed plasma (average of 250 mL per bag) or two units of RBC and two units of Lyoplas (200mls when reconstituted). All sites carried a Golden Hour BoxTM (Pelican BioThermal, MN, USA) prehospital that maintains a steady-state temperature of 4 °C (± 2 °C) for 48–72 h.

All services recommended that patients were transfused blood if there was: a) clinical suspicion of (or confirmed) hemorrhage and b) systolic blood pressure was < 90 mmHg (at any time). In cases without documented non-invasive blood pressure, patients with central pulse only were assumed to have a systolic blood pressure of 40 mmHg, a femoral pulse of 60 mmHg, and a radial pulse of 80 mmHg. Patients in cardiac arrest were described as having no central pulse or heart rate. Pediatric patients with central pulse only were considered to fulfill the criterion for hypotension. The HEMS and in-hospitals major hemorrhage protocols from all participating sites were reviewed for consistency of practice. In hospital, transfusion protocols followed national guidelines [19]. No site used a viscoelastic guided transfusion protocol prehospital.

### Data collection

Patients for all arms were identified by the research and laboratory teams at each site based on blood components issued and transfused prehospital. Once a case was identified, the research team completed a case report form (CRF) using a unique identification number, which was a sequential number for each site. The CRFs contained two parts: Part-1 which collected clinical data for up to 24 h, and Part-2 which collected clinical information from 24 h to 30 days, discharge or death, whichever was first.

Clinical data included information on demographics, incident characteristics prior to hospital arrival (scene arrival time, hospital arrival time) abbreviated injury score (AIS) and injury severity scores (ISS), resuscitative parameters and laboratory test results within the first 24 h of emergency department (ED) arrival, in-hospital transfusion requirement at 24 h, morbidity (ventilator days, hospital length of stay, adverse events), and 24-h and 30-day mortality. Cause of death was collected in a standardized way from all hospitals using death certificate, coroner’s report, or clinical notes (either prehospital or in-hospital). Patients were included even if data points were missing, provided mortality outcome and transfusion data were available.

### Analysis

Counts and percentages were used to summarize the distribution of categorical variables. Mean and standard deviation, or median and interquartile range (IQR), were used for continuous data as appropriate. To account for the dependence between HEMS/hospital and outcomes (clustering), generalized estimating equations (robust standard errors and exchangeable correlation structure) were used to evaluate the association between the different blood transfusion arms and 24-h and 30-day mortality, adjusting for age, patient’s prehospital co-variates (i.e., mechanism of injury, prehospital heart rate, and blood pressure, time from injury to scene arrival, time from injury to hospital arrival). Variable selection for the final model was based on those significantly associated (*p* < 0.05) with outcomes in univariable analysis. Effect modification by mechanism of injury (penetrating vs blunt) was investigated. All tests were two-tailed at the 5% level of significance. Statistical analysis was performed using Stata version 16.1 (StataCorp LLC, Texas, USA).

## Results

**Fig. 1:**
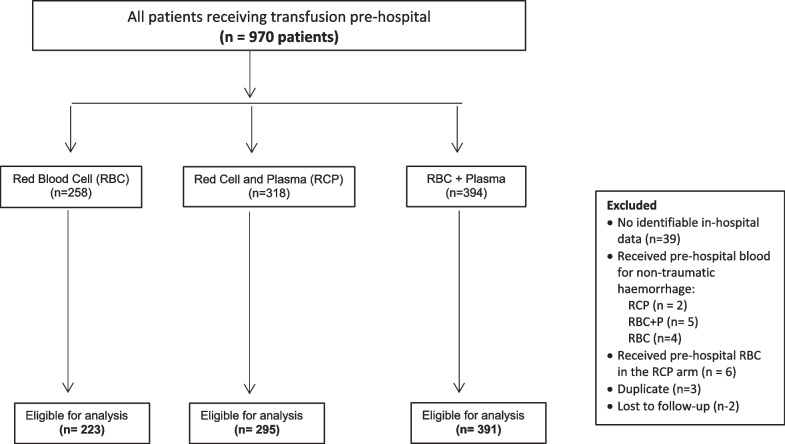
Flowchart of study recruitment

A total of 970 patients were recruited to the study: 258 patients in the RBC arm, 318 in the RCP and 394 in the RBC + P arm. Of these, 61 patients were excluded (see Fig. 1 for reasons for exclusion), leaving a total of 909 patients for analysis. The baseline characteristics for each study arm are summarized in Table 1. Patients in the RBC + P arm were older, had a higher incidence of blunt injury, and had slightly longer median times to scene and hospital. All other covariables, including the injury severity scores (ISS) and prehospital physiology markers, were not significantly different between the three arms.

**Table 1:** Study patient characteristics for different arms

	RBC	RBC + P	RCP
	*N *= 223	*N* = 391	N = 295
	Median (Interquartile Range) unless otherwise stated		
Age, mean (SD)	36.0 (19.3)	42.9 (19.8)	35.6 (19.2)
Male, *n* (%)	178 (80.5%)	287 (75.5%)	244 (83.8%)
Time from injury to scene arrival (mins)	22.5 (14.3, 25.2)	31.4 (26.3, 36.8)	24.6 (14.8,26.1)
Time from injury to hospital arrival (mins)	79.3 (64.2, 89.7)	97.4 (75.2, 107.4)	81.5 (67.4, 91.2)
Blunt Injury, *n* (%)	124 (55.6%)	304 (80.0%)	151 (51.9%)
Injury Severity Score*	33.7 (24, 41)	31.6 (23, 44)	30.1 (22, 43)
AIS Head > 3	27/100 (27%)	51/217 (24%)	38/180 21%
Prehospital physiology			
*SBP (mmHg)*	82.0 (68.0;95.0)	78.0 (67.0;90.0)	79.0 (59.0;100)
*HR (bpm)*	121 (110;133)	110 (81.0;130)	109 (85.5;135)
*HR 0 and SBP 0, n (%)*	55 (25%)	81 (21%)	89 (31%)
*SBP* < *90, n (%)*	198 (88%)	341 (89%)	243 (84%)

Resuscitative and coagulopathy markers at hospital arrival			
	*N* = 87**/158^*†*^	*N* = 178**/285^*†*^	*N* = 194**/214^*†*^
*INR* > *1.2, n (%)*	29 (38%)	52 (30%)	39 (29%)
*Base Excess (mmol/L)*	− 8.00 (− 12.88;− 4.93)	− 5.60 (− 9.90;− 2.42)	− 7.80 (− 14.12;− 3.00)
*pH*	7.16 (7.01;7.27)	7.24 (7.12;7.32)	7.17 (7.01;7.29)
*Lactate (mmol/L)*	4.90 (2.80;8.70)	4.10 (2.40;7.00)	6.50 (3.30;11.2)

*calculated for those eligible as per TARN criteria/from clinical notes

**Number of patients underwent this investigation

^*†*^number of patients who survived to hospital

*ED* emergency department’; *HR* heart rate; *SBP* systolic blood pressure; *INR* international normalized ratio; *ISS* injury severity score; *AIS* abbreviated injury score

Prehospital, patients received a median of two RBC units in the RBC arm (Interquartile Range [IQR] 2;4), one RBC (IQR 0;1) and one plasma (IQR 0;1) in the RBC + P arm, and 2 units of RCP in the RCP (IQR 1;2) arm. Overall, of those that survived to hospital, those in the two plasma arms had a lower incidence for sepsis (chi-square, *p* = 0.041) and received fewer blood components in the first 24 h and after arrival in ED when compared with the RBC arm. Most of the transfusion occurred in the first 24 h across the three arms, with very few transfusions (mainly red cells) occurring beyond 24 h. The median [IQR] of red cells transfused after 24 h was 2 (0.0; 4.0) for RBC arm, 1.00 [0.0;3.0) for RBC + P and 0 [0.0;2.0] for RCP. Survival at 24 h and 30 days for the three arms is provided in Table 2. There was no significant difference in other complications between the three arms (Table 2). The overall causes of death are provided in Table 2, with the breakdown of cause of death at 24 h and 30 days provided under Additional file 1.

**Table 2:** Outcome by prehospital transfusion strategy

	RBC	RBC + P	RCP
	N = 223	*N* = *391*	*N* = *295*
	N (%), unless otherwise stated		
*Mortality†*			
Died on scene	65 (29.1%)	96 (25.2%)	77 (26.5%)
24-h mortality	106 (47.5%)	139 (36.1%)*	117 (40.2%)**
*30-day mortality*	120 (53.8%)	191 (49.1%)	148 (50.1%)
Cause of death			
*Hemorrhage (including tamponade)*	55 (45)	80 (42)	59 (40)
*Traumatic Brain Injury*/Hypoxic brain injury	52 (43)	68 (36)	57 (39)
*Multi-organ dysfunction syndrome*	13 (11)	40 (21)	30 (20)
*Others*	–	3 (2)	2 (1)
Complications			
*Cardiac arrest (in-hospital)*	27 (12.1%)	28 (7.3%)	36 (12.4%)
*Hemorrhagic CVA*	5 (2.2%)	18 (4.7%)	4 (1.37%)
*Transfusion reaction*	0 (0.00%)	1 (0.2%)	0 (0.00%)
*Venous thrombotic event*	3 (1.3%)	15 (3.90%)	15 (5.15%)
*Arterial thrombotic event*	0 (0.00%)	4 (1.0%)	2 (0.69%)
*ICU admission*	106 (47.5%)	187 (49.1%)	166 (57.0%)
*Ventilator days (median, [IQR])*	2 [1;4]	5 [2;8]	3 [1;5]
*ARDS*	4 (1.79%)	4 (1.04%)	1 (0.34%)
*Multi-organ failure*	13 (5.8%)	34 (8.9%)	25 (8.6%)
*Sepsis*	40 (17.9%)	42 (11.0%)	39 (13.4%)
*Nr of days in Hospital, (median, IQR)*	14 (11–17)	13 (9–18)	10 (4–14)

Prehospital blood transfusion			
	*Median (Interquartile range)*		
*RBC (units)*	2 (2;4)	1 (0;1)	**–**
*FFP (units)/Lyoplas*	**–**	1 (0;1)	**–**
*RCP (units)*	**–**	**–**	2 (1.;2)

Blood transfusion from Hospital arrival up to 24 h			
	Median (Interquartile range)		
*RBC (units)*	5 (2;10)	3 (1;6)	4 (2;8)
*Cryoprecipitate (pools)*	1.7 (0;2)	1.1 (0;1.5)	2 (0;3)
*FFP (units)*	4 (2;8)	3 (1;5)	4 (1;8)
*Platelets*	1.1 (0,2)	0.75 (0, 1)	0.94 (0,2)

^*†*^*24 h mortality includes those who died on scene and 30-day mortality includes those who died at 24 h*

*5 unknown

**4 unknown

*RBC red blood cells; P plasma; RCP red cell and plasma combined; CVA cerebrovascular accident; ICU Intensive Care Unit; ARDS adult respiratory distress syndrome; IQR Interquartile range, FFP fresh frozen plasma*

Multivariate regression analysis using generalized estimating equations (adjusting for age, mechanism of injury, prehospital heart rate and blood pressure) showed that compared to RBC, the RCP arm and RBC + P arm were associated with lower odds of death at 24 h (adjusted odds ratio [OR] 0.69 [95%CI 0.52;0.92] and 0.60 [95%CI; 0.32; 1.13], respectively). Investigating effect modification by type of injury showed that lower odds of death at 24 h for RCP and RBC + P arms were driven by penetrating injury (OR 0.39 [95%CI: 0.20; 0.76] and 0.22 [95%CI 0.10;0.53], respectively) and not blunt injury (OR 1.03 [95%CI: 0.75; 1.41] and 0.94 [95%CI 0.48;1.86], respectively). Additionally adjusting for time to scene and time to hospital arrival did not materially alter results and thus was omitted from the final model. (Table 3). For 30-day mortality, the multivariate regression analysis which adjusted for the same co-variates as the 24-h mortality analysis showed no association between RCP (OR 0.84 [95%CI; 0.59; 1.21]) or RBC + P (OR 1.00 [95%CI: 0.72; 1.38]) with survival, compared with RBC alone.

**Table 3:** Association between different treatment and 24-h mortality

Model 1	CRUDE			
	OR	95%CI		P value
RBC	1			
RBC + P	0.39	0.20	0.77	0.006
RCP	0.74	0.57	0.95	0.021

Model 2	Adjusted for injury (no interaction), age, HR & SBP			
	OR	95%CI		P value
RBC	1			
RBC + P	0.60	0.32	1.13	0.116
RCP	0.69	0.52	0.92	0.012
*Injury*				
All Blunt	1			
All Penetrating	0.72	0.50	1.04	0.08

Model 3	Adjusted for injury, age, HR & SBP + interaction between treatment and injury			
*Blunt*				
RBC	1			
RBC + P	0.94	0.48	1.86	0.863
RCP	1.03	0.75	1.41	0.848
*Penetrating*				
RBC	1			
RBC + P	0.22	0.10	0.53	0.001
RCP	0.39	0.20	0.76	0.006

*OR odd ratios; CI confidence interval; N number; HR heart rate; BP systolic blood pressure; RCP red cell and plasma combined; RBC red blood cells; P plasma*

## Discussion

The main objectives of this study were to evaluate the effect of prehospital RCP transfusion (RCP arm) on 24-h mortality and 30-day mortality compared to prehospital RBC transfusion only (RBC arm) and prehospital RBC plus plasma transfusion (RBC + P arm). Our results showed that prehospital plasma transfusion was associated with lower odds of death at 24 h compared to RBC alone (after adjusting for type of injury, age and prehospital observational markers) and that addition of plasma to RBC resuscitation (versus RBC only) brought greater benefit for penetrating injury than blunt injury. At 30 days, there was no difference in survival between the three arms, indicating that the effect of plasma transfusion is greatest in the short term, but there is potential for residual confounding in longer-term outcomes.

The concept of providing damage control resuscitation to trauma bleeding patients shifted to the prehospital environment following publication of the PROPR trial [2] and several observational studies showing survival benefits in patients who received transfusion at the scene of injury [1, 6]. This body of evidence changed prehospital resuscitation practice in the UK, and by the time we started this study, most services had moved to providing RBC and plasma prehospital, meaning that we could not include a prospective RBC arm in our study (a limitation). During the same period, several RCTs were published in this field, aiming to establish the role of RBC and plasma transfusion in prehospital resuscitation. Currently, the role of plasma transfusion in addition to RBC in prehospital hemostatic resuscitation (versus RBC) is poorly understood and remains an ongoing discussion [12].

Two RCTs (PAMPer and COMBAT) that evaluated the effect of prehospital plasma transfusion on 24-h and 30-day mortality provided conflicting results, with PAMPer showing overall survival benefits with prehospital plasma transfusion [9], while COMBAT showed no difference in survival [8]. In this study, we saw that addition of plasma to RBC transfusion prehospital was associated with lower odds of death at 24 h (compared to RBC alone), which is similar to the PAMPer result [9]; however, unlike PAMPer, we saw no association with addition of prehospital plasma to RBC transfusion on 30-day mortality. Previous studies [6] that have evaluated the impact of RBC only transfusion versus saline resuscitation in prehospital setting and the REPHILL trial [7] have also shown a similar pattern of survival benefits with transfusion improving early survival, but having no impact on overall survival. A systematic review on the use of prehospital blood product resuscitation in trauma also showed no overall survival benefit, but there was some evidence for improved survival at 24 h [20]. These results may not be surprising, as from the recent analysis of RCTs, we know that approximately 75% of deaths from bleeding occur within 6 h of injury or hospital admission [21], and beyond 24 h traumatic brain injury or multiorgan failure with sepsis is the main causes of deaths [22].

The post hoc analysis of PAMPer and COMBAT trials showed that the benefit from plasma is primarily seen in blunt injury patients who had a transport time of > 20 min [13]. The results of this study appear contrasting, although caution is needed when comparing results, as the fundamental differences in study designs, population and methods make comparisons difficult. Firstly the ‘standard of care’ arm in the COMBAT trial was 0.9% saline [8], while in PAMPer trial, it was either RBC transfusion (13 of the 27 air medical services), or crystalloid-based resuscitation only [9]. The PAMPer trial had higher rates of blunt injury (73% control vs 81% intervention), which is similar to the RBC + P (80%) arm, whereas penetrating injury was predominant in the COMBAT trial (54% intervention vs. 47% control), comparable to the RBC (45%) and RCP (48%) arms of this study. The typical transport time in the COMBAT study was 16–19 min, while in the PAMPer trial, it was 39–52 min. In this study, the transport time was longer for all three arms with the median time ranging between 80 and 97 min. The injury severity scores (ISS) of patients in PAMPer (median 22) and COMBAT (median 27) trials are lower than in this study (median 30 to 33), and indeed, this reflected the overall differences in baseline mortality at both 24-h and 30-day between studies. These sources of variation could explain the differences in results between studies.

One major area of future prehospital research should be to better identify the group of patients that are most likely to benefit from prehospital plasma transfusion. Understanding the coagulopathy of different types of injuries and the mechanism whereby plasma transfusion corrects these coagulopathies is key to addressing this important topic [12]. In addition to providing clotting factor replacement, plasma is also an ideal volume expander in the intravascular space and several in vitro studies have now shown that plasma also has a homeostatic effect on endothelial function and innate immune system activation, leading to an improved inflammatory response to injury compared with crystalloid resuscitation [23–26]. Further analysis of the COMBAT study showed that prehospital plasma resuscitation reduces the incidence of hyperfibrinolysis [27]. We were not able to measure this in our study, but it is a possible mechanism by which early plasma transfusion corrects the coagulopathy of penetrating injury patients and better diagnostic tests are required to prove this.

While these data suggest that plasma has benefits on coagulation and endothelium, there remain logistical challenges with providing prehospital blood transfusion. The main advantages of administering a combined component like RCP or whole blood, versus separate components, are the additional logistical benefits on scene, such as shorter transport time to hospital and the reduced number of processes, personnel and resources required to administer blood prehospital. Based on the results of this study, these advantages were not translated into clinical benefits for the RCP arm, and this is likely due to the same transfusion content of prospective arms in this study (i.e., RCP and RBC + P) and any potential benefits (if these exist) from shortening the prehospital transport time might be too small to detect given the current sample. Either way, our results show that the clinical benefit from prehospital plasma transfusion appears to be consistent, irrespective of the mode of transfusion (separately or combined), which may give reassurance to services deciding on the best plasma component/product to use prehospital [28]. If the results of upcoming whole blood trials show no benefits with a whole blood component in prehospital environments, then the RCP component could become the frontrunner for prehospital services in the UK to treat traumatically injured bleeding patients, considering its logistical benefits and equivalent effect on outcomes with current standard practice. In this scenario, extending its shelf life to beyond 14 days would be a priority for reducing hospital blood wastage [18, 29].

### Strengths and limitations

A key strength of the study is that the data for plasma arms were prospectively collected by several air ambulance services in England, with information being directly retrieved from patients’ case notes and not from administrative databases. Further, the design of the study did not rely on patient consent for data collection, a potential source of selection bias. Some limitations, however, should be recognized. Firstly, this was an observational study, and its conclusions are not as definitive as those from a randomized controlled trial for evaluating a strategy. Secondly, the sample size for the RBC arm was smaller than the other two arms, due to the data being collected from a single site, and furthermore, the data for the RBC arm were collected at a different period compared to plasma arms and it could be argued that overall treatment of bleeding patients would have changed over time, influencing the survival independent of transfusion. However, the similarities in patients’ characteristics and their outcomes between RBC and RCP arms mitigate these concerns. Further, the implementation of RCP transfusion in London happened the day after cessation of RBC transfusion, and during the study period, the London region did not implement any other major intervention in prehospital environments for treatment of bleeding that could have materially impacted survival. Thirdly, there were some demographic differences between arms, with patients in the RBC and RCP arms being younger than the RBC + P arm and having a higher incidence of penetrating injury and a shorter prehospital transit time, all of which could have contributed to survival outcomes independently of the intervention. The analysis adjusted for these confounding factors as well as differences between services and sites, and evidence for the existence of overall benefit from RCP, compared to RBC alone, remained. Lastly, given the long transport times and the high ISS seen in our cohort, it would have been interesting to evaluate the impact of operative versus non-operative management, but this information was not collected.

## Conclusions

In conclusion, prehospital plasma transfusion in addition to red blood cell transfusion (RBC) is associated with lower odds of death at 24 h compared to RBC transfusion alone, while at 30 days, there was no difference in survival, indicating that the effect of prehospital plasma may be greatest in the short term. There is evidence that the addition of prehospital plasma to RBC transfusion (versus RBC only) was associated with greater benefit for penetrating injury than blunt injury, but future randomized controlled trials are needed to confirm these results.

## Supplementary Information

**Additional file 1.** **Table A.** Cause of Death at 24 hours and 30 days.

## Data Availability

Data supporting the results are presented in the article. The datasets used and/or analyzed during the current study are available from the corresponding author on reasonable request.

## Abbreviations

AIS: Abbreviated injury score
CI: Confidence interval
CRF: Case report form
ED: Emergency department
HEMS: Helicopter emergency medical services
IQR: Interquartile range
ISS: Injury severity score
Lyoplas: Lyophilized plasma
MTC: Major trauma center
OR: Odds ratio
RBC: Red blood cells
RBC + P: Red blood cells and plasma separately
RCP: Red cell and plasma combined (in one bag)
WB: Whole blood
UK: United Kingdom
