# Supplementary material for: Association of red blood cells and plasma transfusion versus red blood cell transfusion only with survival for treatment of major traumatic hemorrhage in prehospital setting in England: a multicenter study
Source: Crit Care. 2023 Jan 17;27:25. doi: 10.1186/s13054-022-04279-4 (PMC9847037; doi:10.1186/s13054-022-04279-4)
Supplement: Supplementary file 1 — Additional file 1. Table A. Cause of Death at 24 hours and 30 days. [file 13054_2022_4279_MOESM1_ESM.docx]

**Supplementary material**

**Table A. Cause of Death at 24 hours and 30 days**

|  | **RBC** | **RBC+P** | **RCP** |
| --- | --- | --- | --- |
| **24 hours** | **N (%)** | | |
| *Haemorrhage (including tamponade* | 75 (71) | 97 (70) | 84 (72) |
| *Traumatic Brain Injury* | 31 (29) | 42 (30) | 33 (28) |
| *Multi-organ dysfunction syndrome* | -- | -- | -- |
| **30 days** |  |  |  |
| *Haemorrhage (including tamponade* | 1 (7) | 3 (6) | 1(3) |
| *Traumatic Brain Injury* | 8 (57) | 21 (40) | 14 (45) |
| *Multi-organ dysfunction syndrome* | 5(36) | 27 (52) | 16 (48) |
| *Others* | -- | 1 (2) | 1 (3) |

*RBC = red blood cells; P = plasma; RCP: red cell and plasma combined*
